# Microfabrication of functional polyimide films and microstructures for flexible MEMS applications

**DOI:** 10.1038/s41378-023-00503-5

**Published:** 2023-03-21

**Authors:** Zihao Dong, Qipei He, Dawei Shen, Zheng Gong, Deyuan Zhang, Wenqiang Zhang, Takahito Ono, Yonggang Jiang

**Affiliations:** 1 Institute of Bionic and Micronano Systems, School of Mechanical Engineering and Automation, Beihang University, Beijing, 100191 China; 2 College of Engineering, China Agricultural University, Beijing, 100083 China; 3 Graduate School of Engineering, Tohoku University, 6-6-01 Aramaki-Aza-Aoba, Aoba-ku, Sendai, 980-8579 Japan

**Keywords:** NEMS, Electronic properties and materials

## Abstract

Polyimides are widely used in the MEMS and flexible electronics fields due to their combined physicochemical properties, including high thermal stability, mechanical strength, and chemical resistance values. In the past decade, rapid progress has been made in the microfabrication of polyimides. However, enabling technologies, such as laser-induced graphene on polyimide, photosensitive polyimide micropatterning, and 3D polyimide microstructure assembly, have not been reviewed from the perspective of polyimide microfabrication. The aims of this review are to systematically discuss polyimide microfabrication techniques, which cover film formation, material conversion, micropatterning, 3D microfabrication, and their applications. With an emphasis on polyimide-based flexible MEMS devices, we discuss the remaining technological challenges in polyimide fabrication and possible technological innovations in this field.

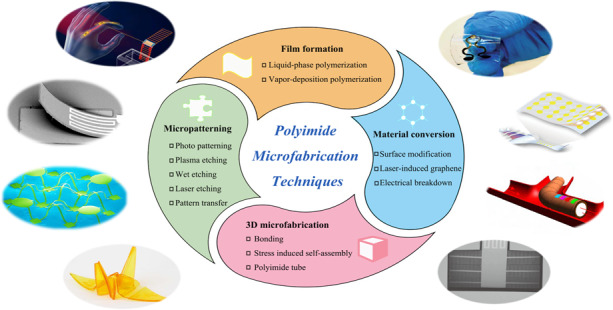

## Introduction

Polyimides have been widely used in the industries of microelectronics, sensors, energy storage, biomedical engineering, and aerospace due to their combined properties, including high thermal stability, mechanical strength, chemical resistance, dielectric properties, and biocompatibility levels. As an important class of commercialized polymers, polyimides are manufactured in the form of films, fibers, foams, composites, and adhesives^1^. For instance, polyimide nanofibers and nanocomposite fibers have various high-value applications, such as gas separation membranes^2^, battery separators^3^, and tissue scaffolds^4^. Since the 1990s, polyimide film has been applied to develop micromachined sensor devices. In 2021, the consumption of PI films reached 2.2 billion dollars worldwide; films are used in various high-tech fields, including solar cells, flexible displays, flexible printed circuit boards, device encapsulation, and flexible sensors^5^.

Unfortunately, there are very few reviews that emphasize the microfabrication techniques of polyimide films. In 1994, Frazier et al. reviewed photosensitive polyimide and plasma etching processes for the fabrication of metallic microstructures^6^. In 1995, Frazier discussed the sensor applications of graphite/polyimide composite materials^7^. In 2007, an engineering review of new materials for microscale sensors and actuators described the processing techniques of polyimides, including wet etching, dry etching, photopatterning, and laser ablation methods^8^. In 2016, Kim and Meng reviewed the development of polymer micromachining technologies, in which polyimide microfabrication strategies were discussed in brief ^9^.

With the rapid progress in microelectromechanical systems (MEMS) and flexible electronics, a series of new fabrication techniques for polyimide films and nanocomposite films have been reported. For instance, the laser-induced graphene (LIG) technique has been well-established for mechanical and chemical sensor applications. The dry etching of polyimide films and stress-induced self-assembly strategies have been widely used in stretchable electronics and flexible 3D microdevices. Thermal bonding and adhesive bonding of polyimide have demonstrated various applications in microfluidics, flexible sensors, and the heterogeneous integration of MEMS. It is necessary to summarize newly developed fabrication strategies with conventional polyimide micromachining knowledge over 30 years.

**Fig. 1:**
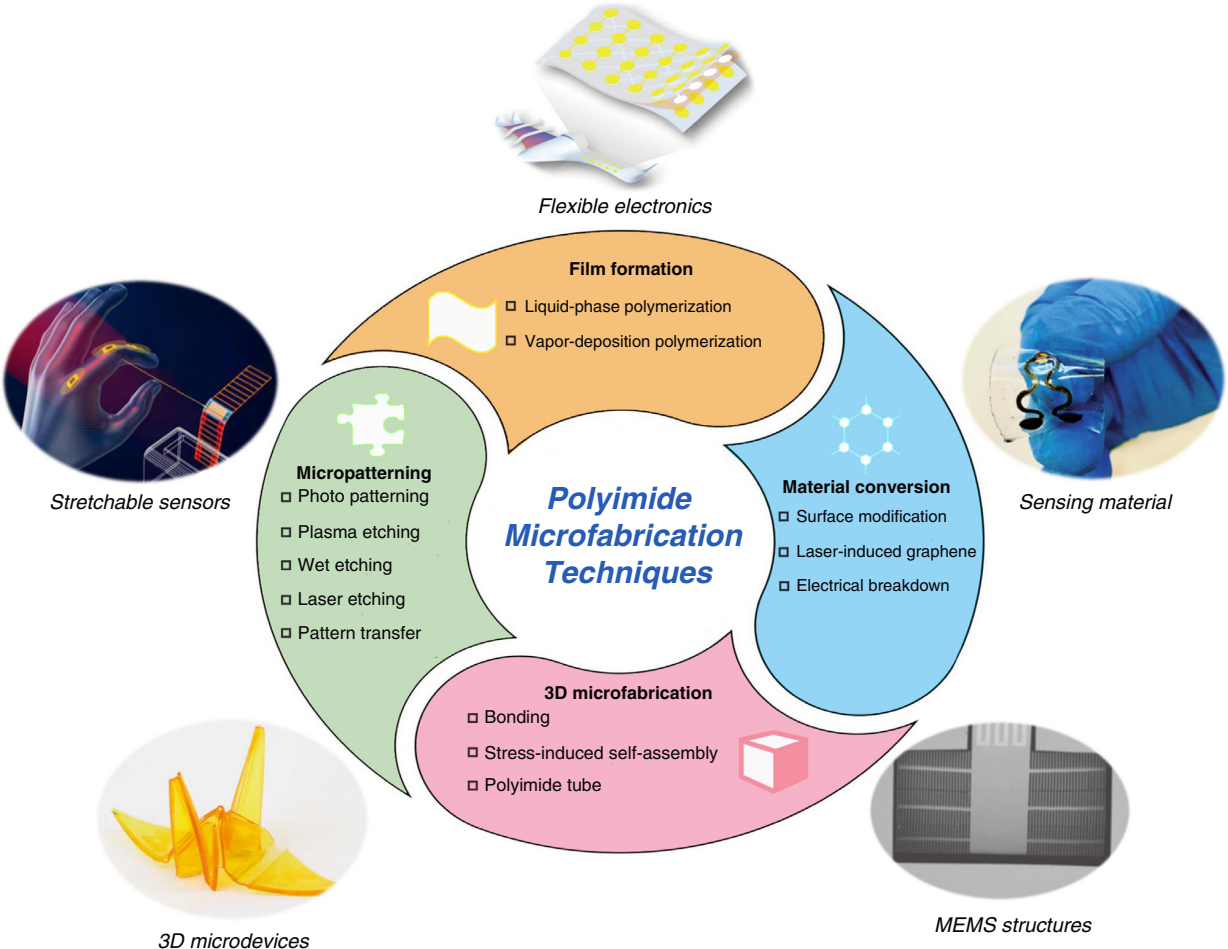
**Polyimide microfabrication techniques and their applications**^77,145,148,166,185^

In this paper, we systematically summarize the essential aspects of polyimide microfabrication that lie behind film formation, material conversion, micropatterning, and 3D microfabrication, as listed in Fig. 1. Furthermore, we highlight the representative applications in MEMS sensors and flexible electronics to enable polyimide microfabrication technologies.

## Formation of polyimide films

### Liquid-phase polymerization

Spin coating, dip coating, and spray coating are all well-established methods for preparing photoresist films. In similar strategies, polyimide can be prepared from poly(amic acid) (PAA) and then thermally cured to thin films. Applications of polyimide coatings include the encapsulation of implantable devices, dielectric layers for 3D packaging, integrated capacitors and the photopatterning of microstructures. As early as the 1990s, polyimide encapsulation has been used in a silicon-based neural electrode array from the University of Utah; polyimide was dip-coated with a primer on the entire surface of an electrode array to provide long-term protection^10^. Spray coating has been used in the deposition of polyimide ablator layers on mandrels^11^ and insulating polymer layers on the surface of a microinjection mold^12^.

Among these liquid-phase polymerization (LPP) methods, spin coating is generally divided into four steps: deposition, spin-up, spin-off, and solvent evaporation. The mechanism of polyimide film formation by spin coating is based on convective flow and solvent evaporation^13^. As polyimide precursors are high molecular weight polymers with high viscosities, their film thicknesses depend largely on the solution concentration, spin rate and time. Unlike traditional photoresists, the thickness homogeneity characteristics of PI films are determined by the spinning and precuring processes^14^. Rubehn and Stieglitz examined the defect density of a spin-coated polyimide film using electrolysis^15^ and proved that a defect-free polyimide layer could be obtained for the highly reliable encapsulation of flexible neural implants.

**Fig. 2:**
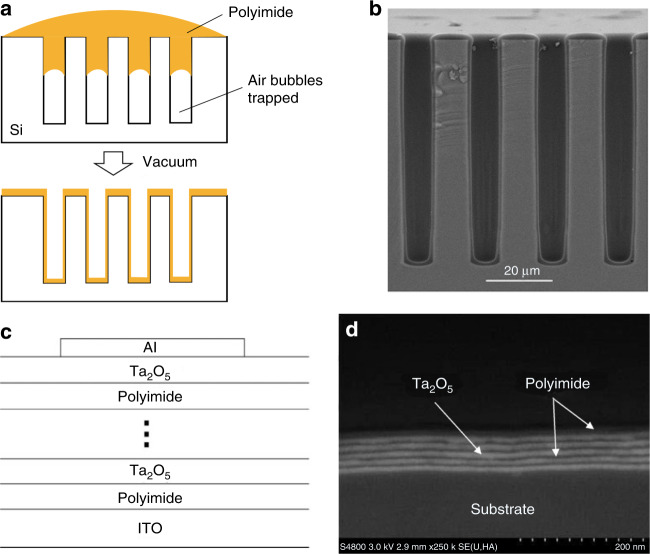
**Vacuum-assisted spin coating and vapor phase deposition of polyimide**. **a** Schematic diagrams of the vacuum-assisted spin-coating technique. **b** Cross-sectional SEM image of the polyimide liner deposition result^18^. **c** ALD process sequence for Ta_2_O_5_/polyimide nanolaminates^34^. **d** Cross-sectional SEM image of Ta_2_O_5_/polyimide nanolaminates obtained via ALD^34^

For advanced packaging and 3D integration applications, vacuum-assisted spin coating techniques and other specialized spin coating methods have been invented for patterning polyimide with good uniformity in deep trenches or high-aspect-ratio (HAR) silicon vias^16,17^, as shown in Fig. 2a. Ding et al. demonstrated a high-uniformity polyimide liner through silicon vias (TSV) with a high aspect ratio of 15:1 (Fig. 2b)^18^. To further improve solvent penetrability for HAR trench filling, supercritical carbon dioxide (SCD) has been employed as a solvent for polyimide polymerization^19^. Using the SCD method, Haruki et al. demonstrated the complete polyimide filling of a silicon trench with a width of 1.5 μm and a depth of 23.5 μm^20^.

### Vapor deposition polymerization

Although LPP techniques have advantages regarding their process simplicity and low cost, they involve solvents and require postdeposition cures, which limits the selection of materials and substrates^21–23^. Moreover, it is very challenging to achieve conformal coatings in microtrenches and other convoluted device structures with these techniques. Vapor deposition polymerization (VDP) is an alternative method for organic synthesis by delivering monomers to a surface during the vapor phase. VDP films are smoother, have better conformal coverage and lower porosities than LPP films. The VDP of polyimide through the vacuum coevaporation of pyromellitic dianhydride (PMDA) and oxydianiline (ODA) was first reported by Iijima et al. ^24,25^ in 1985 and by Salem et al. ^26^ in 1986. PMDA and ODA are evaporated from two separate source ovens with typical evaporation temperatures of 125–145 ˚C and 110–125 ˚C, respectively^27^. The uncured VPD film consists of oligomers and unreacted monomers, while the postcured VDP film is chemically identical to commercially available LLP polyimides. This approach has been applied to BPDA(3,3’,4,4’-biphenyltetracarboxylic dianhydride)–PPD(p-phenylenediamine)^27^, PMDA–DDE (4,49-diaminodiphenyl ether)^28^, and PMDA–DADD (1,12-diaminododecane) films^29^. VDP polyimide (PMDA–ODA) spherical shells are deposited; these shells display better tensile properties and lower permeabilities than spin-cast PMDA-ODA polyimide spherical shells^30^. VDP polyimide-coated carbon fiber composites have higher average tensile strengths than prepared carbon fiber bundle composites^31^. In addition to the vacuum VDP method, the atmospheric pressure chemical vapor deposition of polyimide was explored by González et al. ^32^. The difficulty in this process lies in diminishing crystallized monomers on the surface.

Atomic layer deposition (ALD) is a variant of chemical vapor deposition prevailing in microelectronics industries for high-quality thin film formation on large-area substrates or deep trenches. Putkonen et al. demonstrated the self-limiting deposition of different polyimide thin films at 160 ˚C by using an acid anhydride and a variety of diamines as the second precursor^33^. The imide bond forms in the as-deposited films without any additional thermal treatment, which indicates the ALD growth of the polyimide films. In recent years, polyimide ALD was applied to the deposition of functional nanolaminates, such as polyimide/Ta_2_O_5_^34,35^ and polyimide/AlF_3_^36^. Based on a strategy for alternative nanolayer formation (Fig. 2c), polyimide/Ta_2_O_5_ nanolaminate was successfully deposited, as shown in Fig. 2d.

## Modification and material conversion

### Surface modification

Due to the hydrophobic properties of polyimide films^37–39^, surface modification of a polyimide substrate is usually required to ensure the continuous and uniform deposition of functional materials and robust bonding with other layers. Surface modification methods vary from plasma radiation^40,41^, ion radiation^42,43^, UV/ozone exposure^40,44^, acid treatments^40,45^ and/or base treatments^46,47^.

Plasma radiation is a conventional method for polyimide surface treatment. Kim et al. investigated the adhesive properties of the Cu/Cr/PI system by changing the surface treatment conditions using oxygen plasma^48^. XPS spectra show that the ratio of oxygen and the contents of C-O and C = O functional groups increase on the plasma-treated surface. Surface roughness contributes to improvements in peel strength due to the increased interfacial area caused by plasma etching. Usami et al. conducted microwave plasma treatment on a PI membrane to improve the adhesion between Cu and PI layers^49^. The Ar/N_2_ plasma treatment improved the Cu adhesion force to 10 N/cm even for PI substrates with absorbed water. Atmospheric pressure plasma (APP) has been developed to operate at near-ambient temperatures. Akram et al. investigated the effects of surface modification on polyimide by APP treatment under different exposure times^50^ and observed that the surface energy of the polymer increases with increasing exposure time.

Ion implantation is an alternative surface modification method. Shin et al. investigated the effects of nitrogen ion implantation on the surface microstructures and properties of PI films^42^. Nitrogen ion implantation breaks chemical bonds due to random collisions of incident ions and energy transfer to polymer atoms, and it roughens the surfaces of PI films. Hong et al. reported a plasma source ion implantation technique for improving the adhesive properties of Cu on PI films^51^. A PI film is immersed in plasma, and high negative voltage pulses are applied to accelerate ions into the substrate, resulting in the mixing effect of copper atoms with polyimide layers.

UV/ozone treatment is a very simple process for modifying the wettability characteristics of polyimide surfaces^40,44^; this method does not require complicated vacuum equipment. Koike et al. applied high concentrations of ozone gas (>20%) to modify polyimide films^52^. After exposure to ozone gas, the water contact angle on the polyimide surface decreases from 60° to nearly 20°. To improve the adhesion of the polyimide to the printed Cu electrode, Lee et al. modified polyimide film surfaces to be more hydrophilic by UV/ozone treatment^53^. Enhanced wettability is confirmed by the decreases in the contact angles of the Cu ion complex from 57° to 32° after exposing polyimide to ozone gas for 1 h.

PI surfaces treated with bases and acids convert imide groups to oxime groups. This wet chemical treatment improves the adhesion between polyimides and various polymers and metals. Huang et al. studied the surface modification of Kapton polyimide film by alkaline hydrolysis with KOH aqueous solution^46^. The polar component of the surface energy of the Kapton film increases dramatically within the first minute of KOH treatment. Significant etching occurs for the polyimide film in the alkali solution after a prolonged time. Park et al. modified the surfaces of polyimide films by ethylenediamine treatment to improve their adhesion to a subsequently deposited copper layer^54^.

### Laser-induced graphene

Graphene has many inherent properties, such as high carrier mobility, transparency, flexibility, electrical conductivity, and mechanical strength. Pyrolysis of polymers by scalable laser direct writing (LDW) has provided an effective strategy for preparing graphene^55^. Since the invention of the laser-induced graphene (LIG) process^56^, LIG on polyimide substrates has progressed rapidly with the application of flexible electrodes, strain, and chemical sensors.

**Fig. 3:**
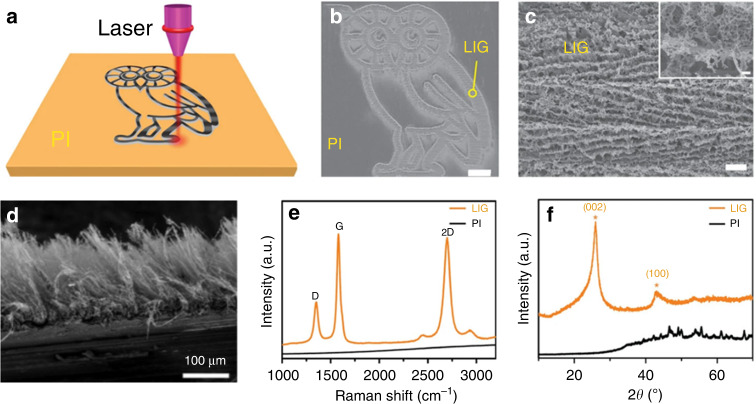
**Laser-induced graphene process**: **a** Schematic diagram of laser-induced PI film^57^; **b** SEM image of the LIG surface^57^; **c** SEM image of the LIG section^57^; **d** SEM image of the fibrous LIG bundles^58^; **e** Raman spectra of the LIG film and PI film^57^; **f** XRD of powdered LIG scraped from the PI film^57^

This specific LIG method scans the PI film with a CO_2_ infrared laser under atmospheric conditions. Due to the photothermal effect caused by laser irradiation, C = O and N-C bonds form. Then, the bonds are broken and the remaining carbon atoms are recombined; the resulting black substance is called LIG (Fig. 3a). Under computer control, LIG can be patterned into various shapes (Fig. 3b), providing a simple approach for manufacturing printable electronics. The LIG exhibits a porous structure (Fig. 3c); the pore size is tunable by controlling the laser power. Due to pulsed laser radiation, the local temperature increases to approximately 2500 ˚C, and the sp3 hybridized carbon atoms in the PI film are photothermally converted into sp2 hybridized carbon atoms, increasing the conductivity^57^. Materials with thick porous structures can be prepared as graphene forests composed of dense long fibrous LIG bundles (Fig. 3d)^58^.

This black substance is known to have a high concentration of graphene. An increased ratio of I_G_/I_D_ in the Raman spectrum and the peak in the XRD curve reflect the high crystalline state of graphene (Fig. 3e, f). Relevant studies have shown that the LIG process exhibits the advantages of high specific surface area, thermal stability, and electrical conductivity^59^. Since porosity is related to the release of gas during conversion, generally increasing the laser power accelerates gas release, increasing the pore size distribution and porosity. In addition, the CO_2_ laser in the preparation process can be extended to new ranges, such as those of ultraviolet lasers and 405-nm visible light^60,61^.

### Electrical breakdown

Electrical breakdown (EBD) of insulating polymers under excessive electrical bias is a well-known phenomenon. Once the electrical current through nanocomposites is well controlled, the electrical breakdown is reversible or irreversible^62,63^. Simoes et al. demonstrated that the EBD voltage of a nanocomposite decreases with increasing filler concentration^64^. Diaham et al. reported the formation of breakdown craters with central punctures in polyimide films after an irreversible EBD process^65^. Therefore, the EBD process is considered a facile method for material conversion.

**Fig. 4:**
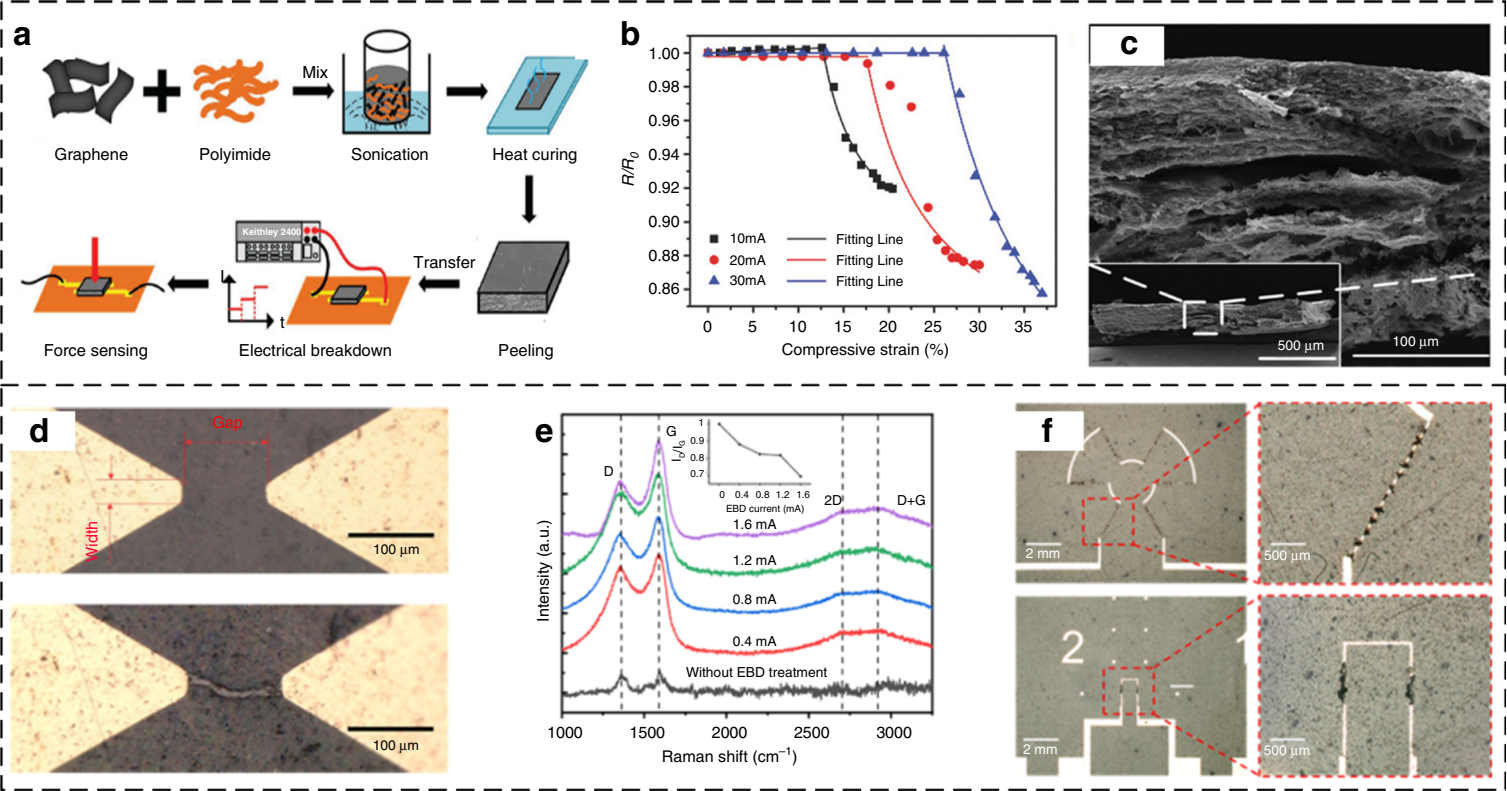
**EBD process**. **a** Fabrication processes of force-sensitive G/PI nanocomposites^66^. **b** Relative-resistance changes in G/PI nanocomposites prepared at different EBD currents^66^. **c** Cross-sectional SEM images of G/PI nanocomposites prepared at an EBD current of 30 mA^66^. **d** Optical images of a G/PI strain sensor before and after μEBD treatment^67^. **e** Raman spectra of μEBD film and PI film^67^. **f** Photographs of the μEBD-treated G/PI strain sensors with different piezoresistor patterns^67^

In 2018, Jiang et al. demonstrated the fabrication of a piezoresistive force sensor based on graphene (G)/polyimide (PI) conductive nanocomposites using a simple EBD approach^66^, as shown in Fig. 4a. The EBD process changed the electrical conductivity of the G/PI nanocomposite, endowing the nanocomposite with piezoresistivity (Fig. 4b). Further investigations showed that the compressive stress sensitivity is tuned by varying the EBD current. The cracks grow with increasing EBD current, resulting in different electromechanical properties of the EBD-fabricated G/PI nanocomposites (Fig. 4c).

However, as the EBD current is applied to a bulk G/PI composite, electrical paths are randomly distributed along the thickness direction, and the piezoresistors exhibit large variations in electrical properties. To fabricate microstructured piezoresistors on thin films with excellent consistency, Jiang et al. developed a microscale EBD (μEBD) method^67^ (Fig. 4d). By applying a constant current to the composite PI between microelectrodes, PI is pyrolyzed due to the local high temperature. The D and G peaks in the Raman spectrum indicate the generation of carbon materials (Fig. 4e). The high piezoresistivity is derived from the high porosities of the carbonized conductive traces that are generated by the μEBD process. Three peaks at 1350 (D-band), 1580 (G-band), and 2700 (2D-band) cm^−1^ indicate the existence of carbon materials, such as graphene and graphite. The value of I_D_/I_G_ tends to decrease with an increase in the μEBD current applied on the G/PI sensors, suggesting a decrease in the number of graphite material defects^68^. By arranging Cr/Au electrodes at different locations on the thin G/PI film and applying μEBD current, diverse piezoresistor patterns are obtained (Fig. 4f). Small Cr/Au dots between the electrodes are used to guide the μEBD process along the designed trace.

In 2019, Jiang et al. proposed a novel flexible airflow sensor, which consists of a self-bending polyimide (PI) cantilever^69^, a flexible double-deck PI film substrate, soft electrodes, and graphene/polyimide (Gr/PI) sensing elements. To improve the gauge factors of the sensing elements, the electrical breakdown (EBD) process is employed to endow the Gr/PI nanocomposite with high piezoresistivity. The airflow sensor achieves a detection threshold of 0.5 m/s over a flow range of 0–20 m/s.

### Flexible sensors via pyrolysis of polyimide

#### Strain sensor

**Fig. 5:**
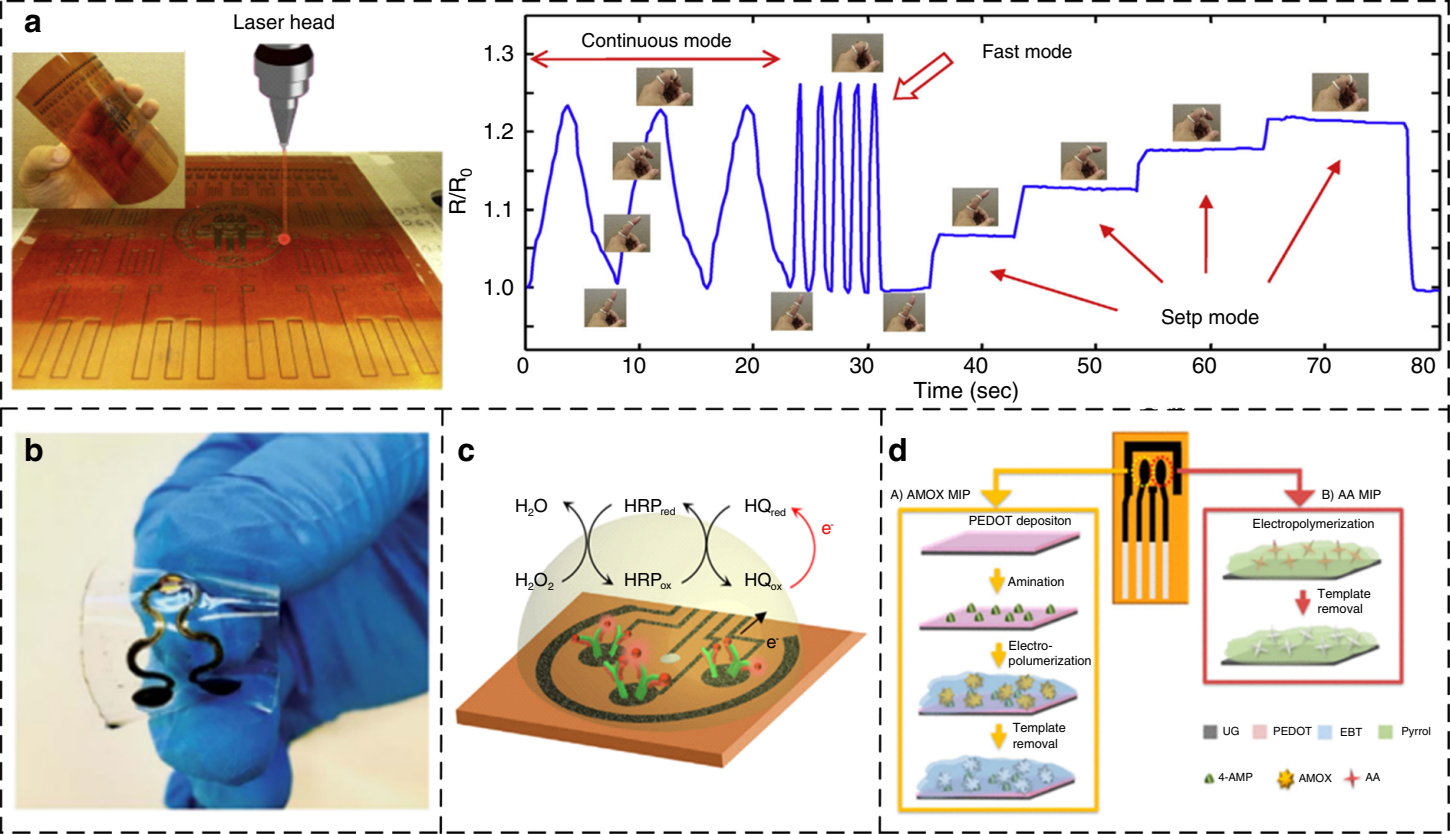
**Flexible sensors via pyrolysis of polyimide**. **a** Patterned LIG sensor on PI film^70^. (**b**) LIG-based NO_2_ sensor^77^. (**c**) LIG-based electrochemical immunosensor array for rapid monitoring of sweat cortisol^78^. **d** Double MIPS sensor based on LIG to detect AA and AMOX^79^

Laser-induced graphene has a porous network structure, exhibiting unique advantages in strain sensing. There are two major contributing factors that generally operate for the piezoresistivity of LIG: (1) the tunneling/hopping effect between neighboring basic structural units (BSUs) due to their distance alteration and (2) the intensive variations, e.g., breakage or loss contact, in the conductive paths in BSU networks^70^. With increasing power, the LIG-generated porous structure softens and weakens, and the distances between neighboring BSUs increase. The structural changes make the hierarchical porous network more easily disrupted when an external load is applied, increasing the piezoresistive response. Since the contact/tunneling resistance has an exponential dependence on the distance separation between neighboring BSUs, higher power leads to higher sensitivity. Luo et al. systematically studied the effects of laser power and scanning speed on the performance levels of LIG piezoresistive sensors (Fig. 5a). The sensitivity of the optimized LIG sensor is nearly 10 times higher than that of a commercial strain gauge^70^. Carvalho et al. developed an LIG strain sensor using a UV laser^60^; the sensor has a spatial resolution that is two times greater than the conventional resolution, and this research provides a strategy for miniaturizing LIG devices. Rahimi et al. reported an LIG-based strain sensor with high stretchability in one direction^71^ by transferring LIG on a PI film to a PDMS substrate. LIG-based strain sensors achieve higher sensitivity levels than those of conductive composite nanomaterial-based strain sensors due to anisotropy.

#### Temperature sensor

Since LIG on a PI substrate has a large negative temperature coefficient of resistance, it is applicable for temperature sensing. In 2017, Marengo et al. prepared a flexible temperature sensor by designing a snake-like temperature sensor on a PI substrate^72^. The resistance of the temperature sensor decreases by approximately 4% when the temperature increases from 22 °C to 58 °C. Bobinger et al. prepared an LIG-based temperature sensor on a PI substrate with a sensitivity of −0.00046 °C^73^. However, when LIG is transferred to a flexible porous PDMS substrate (500 μm), LIG shows a positive temperature coefficient of 0.04 °C; this coefficient, which may be due to the increased LIG resistance resulting from the expansion of the porous PDMS substrate during thermal heating^74^.

#### Gas sensor

Various gas sensors have been developed based on the simple resistance responses of LIG. In 2019, Stanford et al. developed a gas sensor to determine the compositions of gas mixtures (such as N_2_ and CO_2_) based on the LIG Joule heating effect^75^. Ren et al. proposed an LIG-based NO_2_ sensor and self-alarm system (MMSA)^76^. Finger-like LIG and molybdenum disulfide (MoS_2_) were used as the electrode and sensing material, respectively. Additionally, the thermoacoustic effect of LIG is used to generate alarm sound signals when the NO_2_ concentration exceeds 60%. Figure 5b presents a NO_2_ gas sensor based on the self-heating effect of LIG^77^. Taken with the stretchable design layout in the serpentine interconnect region to provide mechanical robustness over a tensile strain of 20%, the gas sensor shows excellent selectivity (6.6 ppm^−1^), fast response/recovery processes, and an ultralow detection limit (1.5 ppb) at a modest temperature.

#### Bioaffinity sensors

LIG-based bioaffinity sensors are manufactured by modifying LIG surfaces using biological receptors, such as antibodies, molecule-imprinted polymers (MIPs), and aptamers. In 2020, Gao et al. developed an LIG-based electrochemical immunosensor to monitor the diurnal dynamics and stress responses of cortisol in biofluids (Fig. 5c)^78^. The key component of the sensor platform is a flexible five-electrode graphene sensor patch fabricated on a polyimide (PI) substrate via laser engraving. Marques et al. achieved the detection of amoxicillin and ascorbic acid with high sensitivity and selectivity by forming LIG on a PI film and modifying MIPs on two conductive LIG working electrodes (Fig. 5d)^79^. MIPs have good binding selectivity levels for target molecules, and they have been successfully applied to analyze binary mixtures in environmental water samples, demonstrating the capability of the device for the analysis of different molecules.

#### Hall sensor

Flexible Hall-effect sensors have been realized in different manners, including by stacked thin films, such as bismuth^80^, permalloy^81^, and graphene^82^, on a flexible substrate. In 2021, Kaidarova et al. reported a flexible Hall sensor based on LIG fabricated by shining a laser on a PI in one step^83^. The Hall sensors offer a linear response to magnetic fields with a normalized sensitivity of ~1.12 V/AT. The sensors exhibit low noise voltage floors of ~50 nV/$\sqrt {{{{\mathrm{Hz}}}}}$for a bias current of 100 µA at room temperature; this result is comparable with state-of-the-art low-noise Hall sensors. The sensors combine a high bendability with a high robustness and high operating temperatures reaching 400 °C.

Carbonized materials generated by pyrolysis PI is widely used as sensing or electrode materials for flexible sensors. By taking advantage of the internal porous structure, high electrical conductivity, good mechanical properties, high thermal conductivity, good surface adsorption, thermoacoustic effect and temperature characteristics of these materials, various physical and chemical sensors have been developed. In these applications, PI substrates and LIG-based sensing materials show the advantages of simple preparation processes, low costs and outstanding sensing performance levels.

## Micropatterning of polyimide

To meet the increasing needs of polyimide microstructures in flexible MEMS, a variety of micropatterning techniques, including photolithography, dry etching, wet etching, laser etching, and pattern transferring, have been developed. In this section, we summarize the mechanisms and technical details of these micropatterning methods.

### Photo patterning

Photosensitive polyimides (PSPI) provide a facile and cost-effective method for the micropatterning of polyimides^84^, which can greatly reduce the number of processing steps relative to the conventional method utilizing photolithography and subsequent etching processes. Notably, PSPI involves photopatternable PI with photoactive agents and photopatternable PI precursors, such as poly(amic acid) (PAA) or poly(amic ester) (PAE) with photosensitive compounds^85^. The PSPI films are selectively exposed to UV light with a mask, allowing the exposed area to undergo a chemical change, such as cross-linking (negative tone)^86–91^ and chain scission (positive tone)^92–98^; undesired areas are dissolved in the developing solution. Methods for incorporating a wide variety of photochemistries into PI systems has been previously reviewed^84,85,99^. In this article, we introduce a few representative photopatterning methods using PSPI.

### Negative-tone PSPIs

Table 1 shows the structure and patterning results of the negative-tone PSPIs, most of which are prepared from PAA. The dissolution rate of PAA in TMAH solution is overly high to obtain a sufficient dissolution contrast between unexposed and exposed areas; thus, photosensitizers are added to the polymer matrix. According to the reaction mechanism, photosensitizers can be divided into two categories: photoacid generators (PAGs)^86,89,90^ and photobase generators (PBGs)^85,88,91^.

**Table 1:** Negative-tone PSPIs

Author	Matrix polymer		Crosslinker	Photo-sensitizer	Feature size (μm)
**PSPI** + **crosslinker** + **PAG**					
Saito et al. ^89^.					6
Watanabe et al. ^86^.					15
Watanabe et al. ^90^.					10
**PSPI** + **PBG**					
Mochizuki et al. ^91^.			–		10
Fukukawa et al. ^101^.			–		8
Tseng et al. ^88^.			–		8
**Photogenerator-free PSPI**					
Chung et al. ^103^.			–	–	10
Li et al. ^102^.			–	–	5

PAG is usually accompanied by a crosslinker, and the photoreaction of PAG produces acid, leading to an acid-catalyzed cross-linking mechanism^100^. Watanabe et al. suggested that the cross-linkers react with each other to form a network structure^90^, decreasing the solubility of the exposed region and forming a negative PSPI. A key role of PBG, which photochemically generates a base compound, is the promotion of thermal imidization to change the solubility between the exposed and unexposed regions in these resists^85^. Fukukawa et al. reported a negative-type PSPI based on PAA and {[(4,5-dimethoxy-2-nitrobenzyl)oxy]carbonyl} 2,6-dimethyl piperidine (DNCDP) as a PBG^101^, identifying effective base catalysts for the solid-phase imidization of PAA at temperatures below 200 ˚C.

The addition of low-molecular-weight additives, such as photoactive compounds and crosslinkers, into a PI system causes losses in thermal and mechanical properties. The photogenerator-free PSPI, which can provide pure PI contents, is an effective solution; however, most of these materials need a process of development with organic solvents^102,103^.

### Positive-tone PSPIs

In negative-tone PSPIs, there is usually a swelling problem, resulting in poor resolution^85^. Positive-tone and aqueous alkaline developable PSPIs are desirable, and their characteristics are summarized in Table 2.

**Table 2:** Positive-tone PSPIs

Author	Photoreaction	Feature size (μm)
**PI structure**		
Shin et al. ^87^.		0.4
Choi et al. ^93^.		1
**PAA** + **DNQ/PAG**		
Yeh et al. ^98^.		3
.Inoue et al. ^96,104^		6
Jung et al. ^94^.		5

By considering the high solubility of PAA in an aqueous alkaline solution, a common solution for avoiding PAA dissolution in an aqueous alkali is capping a carboxylic acid group with an ester group. Then, photosensitizers, such as diazonaphthoquinone (DNQ) and PAG, are added to the system. The mechanisms of DNQ and PAG are different. DAQ is connected with the main chain, and a photochemical transformation of DNQ into indenecarboxylic acid derivative occurs to accelerate dissolution in aqueous alkaline solutions^96,104^. The PAG photoreaction produces acid, which deprotects the acid-labile ester group of PAA^94,98^.

Employing the PI structure as a polymer matrix instead of PAA and derivatives can avoid problematic volume shrinkage in the stage of thermal treatment. Shin et al. reported a positive-tone PSPI that has fully imidized backbones with *o*-nitrobenzylester groups as side substituents^87^, and a lithographic image with a resolution as fine as 0.4 μm is achieved.

### Reactive ion etching of polyimide

Reactive ion etching (RIE) is the most prevalent technology for the patterning of polyimide. Polyimide RIE uses gas mixtures primarily containing oxygen, which is usually named O2–RIE. High directionality, high selectivity, and low surface roughness are the main requirements for RIE. To achieve high selectivity in the patterning of thick PI films, hard-etch masks, such as titanium, aluminum, and silicon dioxide, are commonly used. Relevant studies are summarized in Table 3.

**Table 3:** Plasma and wet etching of polyimide

Author	Etchant	Etching rate (μm/min)	Aspect ratio	Sidewall angle (deg)
**Dry etching**				
Murakami et al. ^105^.	O_2_	4	15	
.Fusao et al. ^106,107^	O_2_ + N_2_	>5	>10	
Bliznetsov et al. ^108^.	O_2_ + CF_4_	3.5		Almost vertical
Zawierta et al. ^109^.	O_2_ + CF_4_ + N_2_	0.86		8
**Wet etching**				
Lin et al. ^184^.	KOH + C_2_H_7_ON	16		
Han et al. ^119^.	TPE3000	5	<1	30
Lena et al. ^121^.	NaOCl	≈0.2	138	
Zuzanna et al. ^120^.	NaOCl	0.05	5.2	15
Kristina et al. ^122^.	NaOCl	0.35	≈6	

### HAR structure preparation

Preparation of HAR structures has been a demanding goal of polyimide RIE. Murakami et al. fabricated HAR vertical structures using an O_2_–RIE system with an aluminum mask, achieving a selectivity of 1000, an etching rate of 4 μm/min, and an aspect ratio of 15^105^. Shimokawa et al. developed a magnetically controlled reactive ion etching (MC–RIE) system. The high oxygen plasma density using MC–RIE achieves a high etching rate (>5 μm/min) and extremely selective polyimide etching with a titanium mask^106,107^. Bliznetsov et al. presented a high-throughput anisotropic RIE method for polyimide in a gas mixture of oxygen and carbon tetrafluoride (CF_4_). By using dual-frequency superimposed capacitively coupled plasma, researchers have achieved a vertical profile of polyimide^108^. The sidewall angle is controlled by varying the chamber pressure at elevated substrate temperatures^109^. In conclusion, the HAR structure is effectively processed by RIE, and the etching rate and directionality are controllable by the process conditions. Additionally, surface roughness and etching residues cannot be ignored.

### Surface roughness control

Surface roughness is another key indicator of RIE for high-quality PI structures. The working pressure is the most important factor affecting the surface roughness. Agarwal et al. found that RIE at a lower pressure facilitates smoother sidewalls; the pressure determines the angles of incidence of the etchant species on the surface^110^. Moreover, the roughness increases approximately linearly with increasing etched depth for a given pressure^111^. Buder et al. revealed that the high surface roughness in the RIE of polyimide is a result of oxygen ion bombardment, which is most pronounced in pure oxygen plasmas^112^. Thus, sulfur hexafluoride (SF_6_) gas is usually added to obtain a smooth etched surface even though it causes a reduced etch rate due to the formation of nonvolatile fluorine compounds inhibiting the reaction between oxygen and hydrocarbon polymers^113^.

### Residue removal

**Fig. 6:**
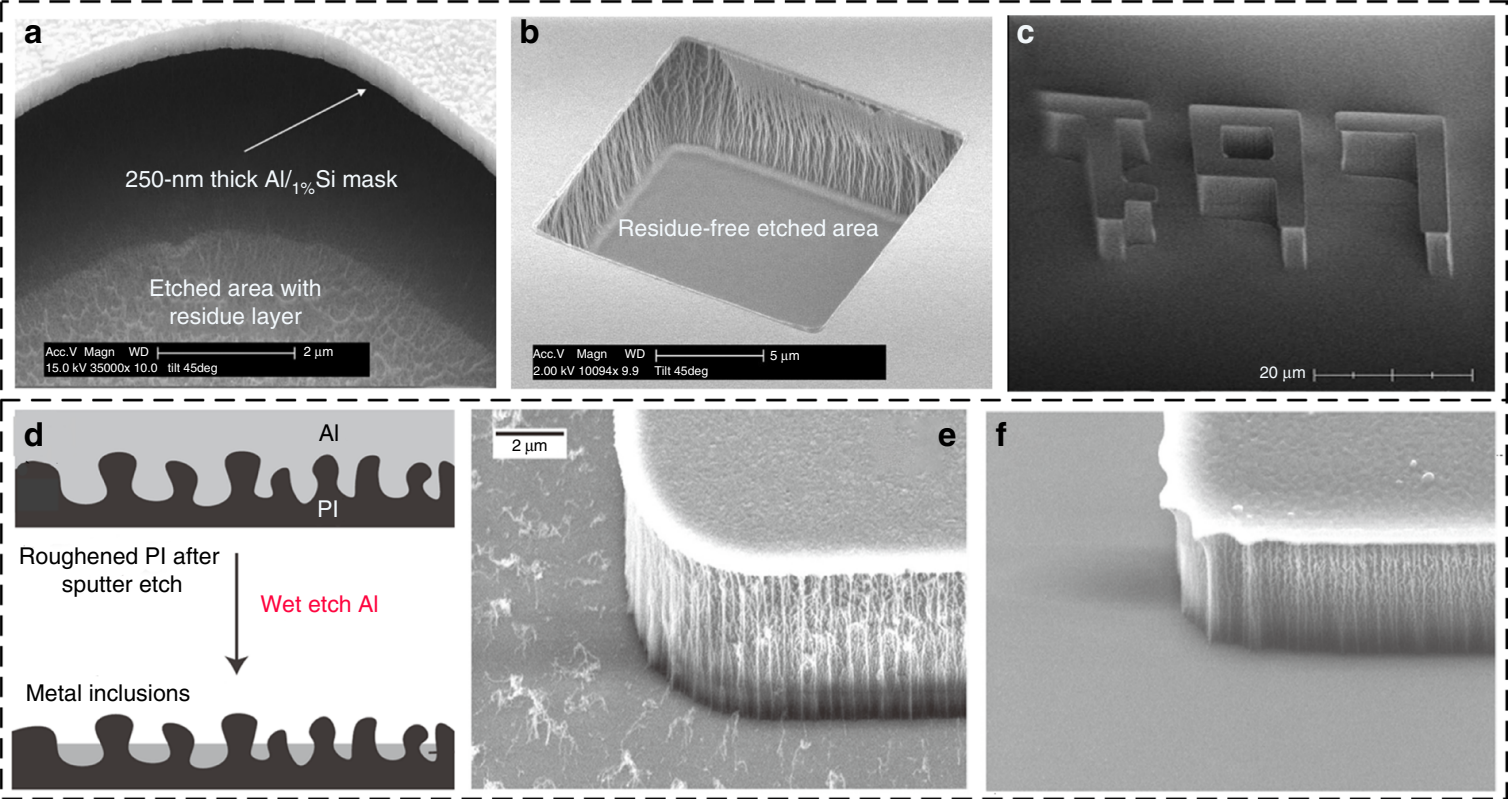
**Patterning of polyimide using dry etching**. **a** Residue layer after isotropic plasma etching of polyimide in pure oxygen^115^. Isotropic (**b**) and anisotropic (**c**) residue-free etched surfaces^115^. **d** Schematic of a rough PI surface with an aluminum layer on top and the metal inclusions inside the roughened pockets of PI that remain after a wet etch^116^. **e** Fur-like residue after PI dry etching^116^. (**f**) Fur-free PI surface with an aluminum mask patterned by dry etching in a Cl_2_ plasma^116^

As traditional polyimide O2–RIE produces residues (Fig. 6a), the removal of residues is crucial for many applications. A simple approach is to use ultrasonic cleaning^114^. Several methods have been proposed to achieve the residue-free plasma etching of polyimide. The addition of a small amount of fluorine-containing gas CF_4_ to the etching mixture may completely remove the residue layer (Fig. 6b, c)^115^. Notably, RIE with hard masks, such as Al, causes fur-like residues^116^. The sputtering etching of the metal mask forms metal inclusions on the roughened PI layer on top (Fig. 6d). These inclusions are not etched in the wet etch of the metal mask because the low surface energy of PI leads to the formation of metal-containing residues (Fig. 6e). Joshi et al. demonstrated that a slight over-etch of 15 s is sufficient for eroding the top layer of the PI and removing metal inclusions by using Cl_2_ plasma, thus enabling a fur-free PI etch (Fig. 6f)^116^.

### Wet etching

Wet etching is an efficient method for removing PI sacrificial layers or residues. Although the wet etching of PI is principally isotropic and the wall topography is hard to control, many efforts have been made to micropattern PI using wet etching (Table 3). The hydrolysis mechanism of PI in a strong alkaline solution (5.3 wt.% KOH) is investigated^117^. The dissolution of PI in alkaline solution includes two steps: the hydrolysis of imide to amide structures and the further hydrolysis of polyamide forms for small species.

**Fig. 7:**
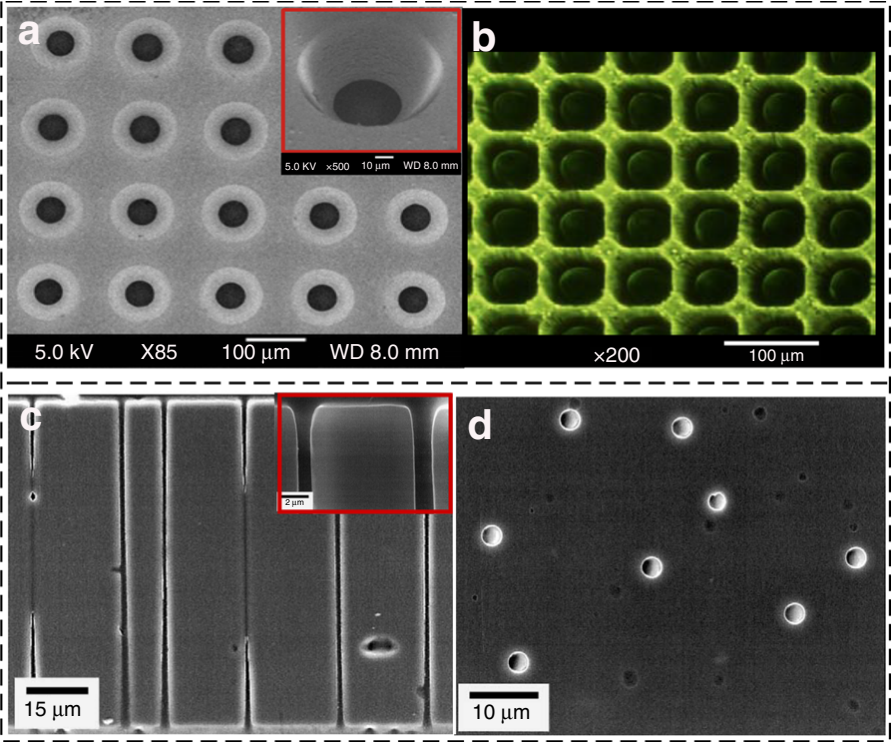
**Wet etching of polyimide**. **a** Arrayed throughholes with pitches of 150 × 150 μm^2^ and (**b**) 70 × 70 μm^2^ fabricated by wet-etching^119^. **c** Cross sections and (**d**) top surfaces of the throughholes fabricated by track-etching^121^

To develop an efficient and facile patterning method, Liu et al. investigated the wet etching of polyimide film in a special alkaline etching solution. The researchers found that an alkaline solution with a concentrated amine etchant, such as ethylenediamine, will result in a high etching rate, whereas alkaline solutions with volatile etchants, such as ammonia or dimethylamine, have a relatively low etching rate^118^. Han et al. fabricated high-density microscale throughholes on a flexible PI film by wet etching. TPE3000 etchant, which is composed of 20 wt.% KOH solution and 20–40 wt.% aliphatic amine compound C_2_H_7_NO, is used in the etching process. Arrayed microscale throughholes with diameters of less than 100 μm and pitches of 70 μm are fabricated on 50-μm-thick PI films (Fig. 7a, b)^119^.

The above isotropic wet etching method has difficulty obtaining structures with large aspect ratios. To achieve the anisotropic etching of polyimide films, a track-etching technique is proposed. The polyimide foil is first penetrated by a single heavy ion (e.g., Au, Bi, U) with a total kinetic energy of several hundred MeV to some GeV; then, the damage is highly selectively etched in sodium hypochlorite (NaOCl) solution^120^. Klintberg et al. studied the development and etching of heavy ion tracks in polyimide with NaOCl and achieved throughholes with a high aspect ratio of 138 (Fig. 7c, d)^121^. The track-etching technique enables the fabrication of low-cost, homogeneous, and highly efficient nanopores on a massive scale^122^.

### Laser etching

Laser etching is a highly versatile technique for micromachining metals, polymers, glass, and ceramic materials. By using the photothermal and photochemical effects of high-intensity lasers, laser etching can be a low-cost and highly efficient patterning method for polyimides.

The direct ablation of polyimide by infrared laser (10.6 μm of CO_2_ laser, 1064 nm of Nd:YAG laser, etc.) has been more extensively studied. In the 1980s, infrared CO_2_ lasers were investigated for polyimide etching via a pure thermal process. Imai et al. demonstrated that a Q-switched CO_2_ laser tuned at 9.3 μm is a promising beam source for the high-speed etching of polyimide films^123^. However, the direct ablation of polyimide generates charred surfaces with black fibrous products^124^ and soot^125^ and causes the thermal degradation of polyimide^126^. A CO_2_ laser tuned to a wavelength that is strongly absorbed by polyimide can remove debris on a polyimide surface^127^. Lim et al. discovered that the rapid outgassing of the pyrolysis gas may push the carbon residuals out from the PI matrix, achieving an unexpectedly smooth surface under specific laser conditions^128^.

Different from the photothermal mechanism and innate debris problem of direct ablation by infrared lasers, ultraviolet (UV) lasers can directly break chemical bonds in polyimide, which is a photochemical decomposition process. This process can precisely remove polyimide material in a geometry defined by the laser beam;^129^ thus, thermal side effects can be avoided^130^. The excimer laser has a broad range of UV wavelengths (157–353 nm), small feature size, and variable pulse width; thus, it is commonly used for the patterning of polyimide^131^. Schammler et al. used an excimer laser with a wavelength of 248 nm for the laser etching of vias and lines on PI films; the sidewalls are straight and sloped between 60° and 85°^132^. In addition to the excimer laser, a solid-state laser is employed in polyimide etching. Pan et al. fabricated microscale hole arrays with different diameters and taper angles from 88° to 82° on a 50-μm-thick PI film^133^. Mullan et al. reported arrays of reproducible holes with entrance diameters on the order of 14 μm and exit diameters on the order of 3 μm in 125-μm-thick PI films^134^.

In recent years, ultrashort pulse lasers with pulse durations on the order of femtoseconds have become powerful and precise tools for polyimide microstructuring^135^. In contrast to conventional laser processing, the intense field created by the focused pulse ionizes the material and forms a plasma to absorb photons directly, resulting in cold ablation without heat affecting the material. Therefore, the structural size is not limited by thermal or mechanical damage; instead, it is determined by the diffraction limit of the optical system used^136,137^. Schwerter et al. structured a polyimide cavity (depth 12–13 μm) using a femtosecond laser with a 515 nm wavelength and a pulse length of 230 fs. By varying the pulse energy, the etch rate may be adjusted from 1–2 μm per pulse to the selective removal of the complete polyimide layer from the carrier substrate^135^. Laser etching has been widely used for the micropatterning of PI films. However, the processing accuracy can hardly reach the levels of photolithography and plasma etching techniques.

### Pattern transfer

Pattern transfer is a facile process for patterning polyimide that avoids complicated photolithography and subsequent etching processes. As the scale of the PI structures prepared by the pattern transfer method depends on the dimensions of the molds, nanoscale structures can be processed.

**Fig. 8:**
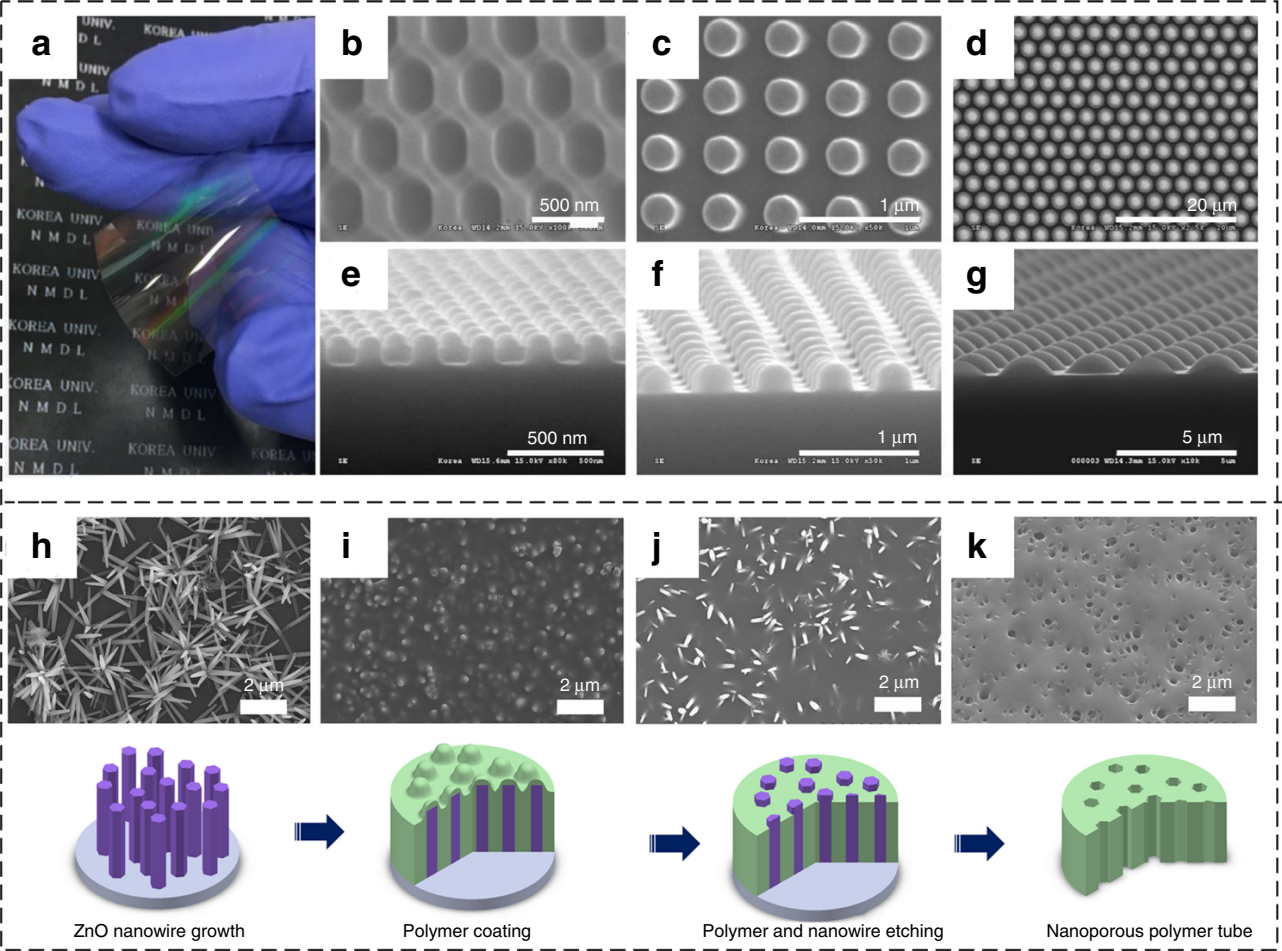
**SEM images and corresponding schematic representations for the nanopatterned polyimide via pattern transfer**. **a** Photograph of patterned PI film on a flexible substrate and (**b**–**g**) SEM micrographs of various microscale and nanoscale PI patterns^139^. **h** Growing ZnO nanowires on a flat glass substrate. **i** Coating the ZnO nanowires with a layer of polyimide, which is then cured. **j** Etching the top layer of the polyimide film. **k** Resulting nanoporous polyimide membrane after etching the nanowires and substrate^141^

Nanoimprint lithography (NIL) is a typical pattern transfer method that has been used for the fabrication of polyimide nanostructures^138^. There are two approaches to pattern polyimide using NIL: (1) imprinting at its uncured soft state and then curing and (2) direct imprinting into polyimide at temperatures higher than its glass transition temperature. By using the first approach, Jun et al. successfully fabricated nanoscale to microscale optical patterns of polyimide (Fig. 8a–g). The PDMS mold is brought into conformal contact with the uncured polyimide substrate and then cured by heat or ultraviolet exposure^139^. Cui et al. successfully fabricated polyimide gratings with a 200-nm period by both approaches. Relative to the first approach, a high-temperature imprint may lead to large thermal stresses, large misalignment, and other unfavorable features^140^.

Another approach is to process an easily removable mold as a sacrificial layer, to coat and cure PI on it, and to then remove the sacrificial layer to obtain a patterned polyimide. An et al. reported the fabrication of nanoporous polyimide membranes with a pattern transfer method using ZnO nanowire arrays as templates^141^. The fabrication process is shown in Fig. 8h–k. First, ZnO nanowire arrays are prepared via a hydrothermal method. Then, a polyimide solution is spin-coated onto the planar substrate on which the ZnO nanowires are grown and cured in a nitrogen atmosphere. To remove the top layer of the polyimide film covering the ZnO nanowires, RIE is performed using a gas mixture of O_2_ and CF_4_. Finally, a nanoporous membrane forms after ZnO nanowires and glass substrates are etched with 6 M NaOH and 10 wt.% HF solutions, respectively.

### Device application of polyimide micropatterning

The patterned polyimide, which is usually used as the sacrificial layer, insulation layer, or structural layer, has a wide range of applications in integrated circuit packaging, flexible electronics, and microsensors.

### Polyimide as a sacrificial layer

Polyimide can be used as a mold or sacrificial layer to build microstructures of otherwise difficult-to-machine materials. Murokami et al. fabricated metal micro gears by a PI sacrificial layer removal process. Electroplated copper structures form in the polyimide molds and are then released by the dry etching of PI^105^. Bliznetsov et al. achieved 0.3-μm-thick and 7.5-μm-high SiO_2_ walls after polyimide patterning and etching SiO_2_ deposited on the PI column; sacrificial polyimide is removed in isotropic O_2_ plasma^108^. Aggarwal et al. presented a novel low-temperature fabrication process that combines polyimide structures with electroless copper plating to create low-stress composite structures for extremely fine-pitch wafer-level packages. Metal-coated polymer structures in conjunction with thin solder bonding films can provide low-cost, high-performance solutions for wafer-level packaging^114^.

### Polyimide as an isolation layer

Polyimide is a reasonable choice for insulating layers and thermal isolation layers in three-dimensional interconnections. Fan et al. investigated a photosensitive polyimide-based insulating layer fabrication for MEMS applications. PSPI is spin-coated on a silicon substrate as an insulating layer between two metal lines^142^. Jang et al. reported a photoinitiator-free photosensitive, soluble polyimide gate insulator that is robust against patterning^143^. In terms of thermal isolation, Fan et al. introduced a method for isolating thermal conduction from a silicon substrate for accommodating thermally sensitive microdevices using a low-stress photosensitive polyimide suspension structure. Due to the excellent thermal isolation performance of PSPI, the suspended PSPI membrane is promising as an outstanding candidate for thermal isolation applications^144^.

### Polyimide as a structural material

**Fig. 9:**
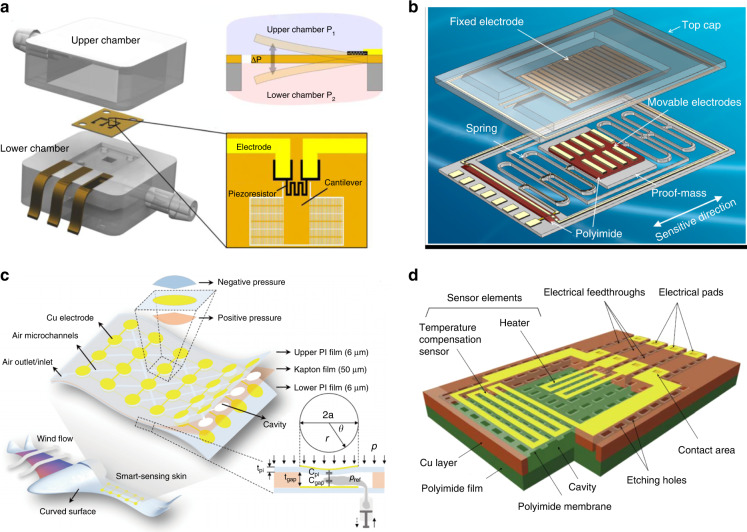
**Device application of a patterned polyimide**. **a** Photosensitive polyimide cantilever-based differential pressure sensor^145^. **b** High-resolution micro accelerometer^146^. **c** Schematic diagram of a UAV equipped with smart sensing skin for pressure sensing^148^. **d** Respiration sensor integrated with a temperature compensation sensor^149^

Polyimide is widely used as a sensing or structural material in flexible MEMS devices because of its excellent flexibility and mechanical properties. Zhao et al. proposed a bristled cantilever-based differential pressure sensor inspired by the bristled wing configuration of tiny insects (Fig. 9a). A novel pressure differential sensor with a bristled PI cantilever is fabricated using PSPI, which can expand the direction range while retaining sensitivity in the low pressure range^145^. To develop a high-resolution micro accelerometer (Fig. 9b), Wu et al. proposed a novel optimization method involving the insertion of photosensitive polyimide to reduce the parasitic capacitance and thus to overcome the fringe effect^146^. Xiong et al. developed a flexible capacitive sensor array with high linear sensitivity for wind flow pressure measurements of unmanned aerial vehicles (UAVs). A polyimide film is cut into an array of cavities by a laser etching process that acts as an intermediate layer (Fig. 9c)^147^. While changing the reference pressure through the microchannels, the sensing range can be customized arbitrarily according to different flight conditions and measured positions without any deterioration of sensitivity^148^. Kato et al. produced a cavity structure for thermal insulation of the flow rate sensor and electrical wiring structures by selectively etching a copper foil from holes formed on the photosensitive polyimide film layer (Fig. 9d)^149^. The researchers have developed a respiration sensor integrated with a temperature compensation sensor on a polyimide substrate and measured the respiration of both rats and rabbits, despite the differences in temperature between exhalation and inhalation.

## Microfabrication of 3D polyimide structures

It has always been a technological challenge to obtain 3D structures using conventional MEMS techniques. In this section, we describe the bonding and self-assembly processes for 3D polyimide structures. Additionally, we discuss the progress made in polyimide tube-based microfabrication technologies.

### Polyimide bonding

Bonding is a traditional MEMS technique for 3D microstructures and device encapsulation applications. During the last two decades, polyimide as an interlayer has been extensively studied for low-temperature silicon wafer bonding due to its low glass transition temperature (Tg) as a well-developed polymer^150–152^. The typical bonding temperature is lower than 300 ˚C, and the applied load is usually lower than 0.5 MPa; the applied load can even be generated by an electrostatic force^152^.

Polyimide-to-polyimide bonding is reported for polyimide microfluidics and 3D flexible sensors. One of the direct bonding strategies is to use a precured polyimide layer by cure cycle adaptation. By the surface treatment of soft-baked photosensitive polyimide with NMP, Metz et al. achieved polyimide-based microfluidic devices with polyimide–polyimide bond strengths comparable to the bulk properties^153^. Notably, the partially cured PI film has still undergone a cross-linking reaction, producing gas molecules and forming voids at the bonding interface^154^. This strategy has been employed in PI–PDMS bonding for the microassembly of PDMS microfluidics with integrated flexible circuits^155^. A microstructured PDMS layer is bonded to a flexible polyimide PCB using a combination of oxygen plasma treatment and chemical bonding with 3-aminopropyltriethoxysilane (APTES)^155^. Another strategy is to use an adhesive polyimide precursor as the bonding layer. For instance, Mangriotis presented the bonding of a fully cured polyimide film to an open channel structure by using a thin layer of polyimide precursor as an adhesive^156^. Kilaru et al. demonstrated a NiCr MEMS tactile sensor embedded between two polyimide layers by an adhesive polyimide strategy^157^.

In the case of low-cost and rapid prototype applications, a heterogeneous interlayer is used for polyimide-to-polyimide bonding. Wang et al. reported a wax thermal-fusion bonding process for the fabrication of a microfluidic mixer, in which the wax layer serves as the mask for polyimide etching^158^. Huang et al. employed epoxy resin as the interlayer to achieve polyimide-based capacitive pressure sensors^159^.

Thermal bonding with a polymer middle layer is a common strategy. Polymers that are suitable as thermal bonding adhesives for polyimide should have a low glass transition temperature than polyimide, high bonding strength, and good mechanical strength. Based on these criteria, Hu et al. used fluorinated ethylene propylene (FEP) and perfluoroalkoxy alkane (PFA) nanoparticle aqueous dispersions as adhesives for multilayer polyimide thermal bonding^129^. In this manner, microchannels are connected vertically between layers to form 3D structures.

### Stress-induced self-assembly of polyimide structures

Although there are many methods for the self-assembly of polyimide microstructures, controlling the stress gradient in suspended microstructures is a well-used strategy for its compatibility with conventional MEMS technology. In this article, we introduce several methods for creating stress gradients, such as bilayer structure, localized activation, and compressive buckling^160^.

**Fig. 10:**
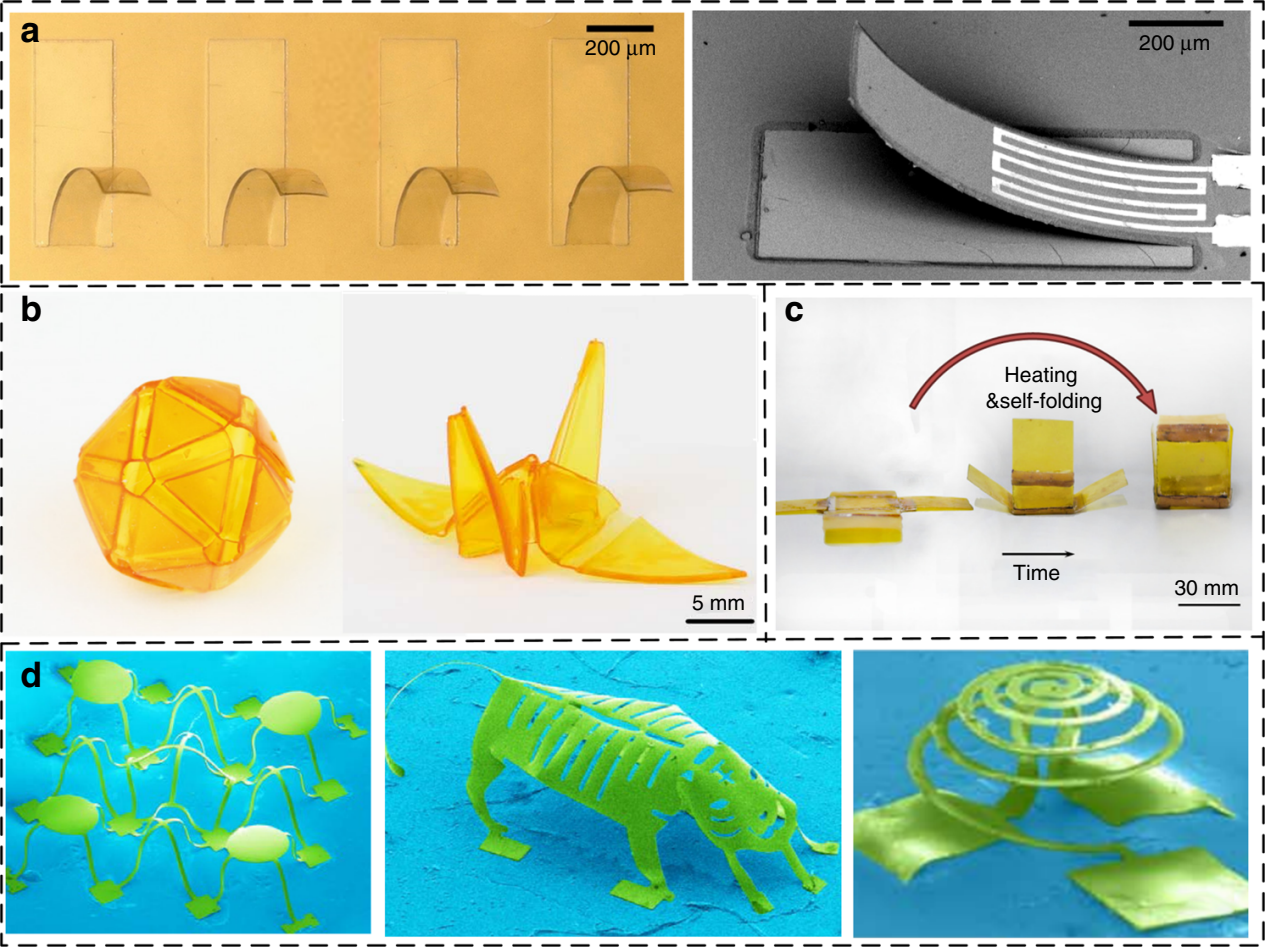
**Polyimide 3D structures**. **a** Bending of the polyimide/silicon cantilever^162^. **b** 3D origami structures by leading the light field to nonuniform curing of the polymer resin^166^. **c** 3D polyimide box through powerful driving force from shape memory polyimide ink hinges^167^. **d** 3D microstructures driven by compressive buckling^171^

A bilayer structure consists of a polyimide and another material with a different coefficient of thermal expansion (CTE). Due to the different CTEs, thermally induced stress causes a uniform curvature in the bilayer structure, which is inversely proportional to the thickness. Watanabe et al. developed a microassembly technique for a 3D polyimide structure using the bending of a polyimide/chromium bilayer cantilever^152^. The contraction of the polyimide film and residual stress of the Cr film induce a bending force. Aiyar et al. presented an out-of-plane polyimide/silicon dioxide cantilever to improve the flow–structure interaction^161^. Shen et al. proposed self-bended 3D hair-like polyimide/silicon curved cantilevers for airflow sensing (Fig. 10a)^162^. In their study, the curvature of the self-bended cantilevers is modified by the thickness of the sputtered silicon layer.

Another approach for fabricating polyimide 3D structures is to introduce a stress gradient by localized activation^163^. Alberto et al. used the laser origami technique to generate larger 3D structures by the controlled folding of Kapton foil due to the volumetric contraction of metallic nano inks placed at strategic locations over the foils^164^. The curing of the nano inks results in the evaporation of the organic solvents and subsequent sintering of the metallic nanoparticles, both of which result in the significant volume shrinkage and consequently out-of-plane folding of the selected components^165^. By adding photosensitive absorbers to the polymer resin, Zhao et al. proposed an approach for creating 3D origami structures by leading the light field to nonuniform curing along the direction of thickness (Fig. 10b)^166^. As the layer directly exposed to light cures faster than the next layer, this nonuniform curing degree leads to a stress gradient. By using a novel shape memory polyimide ink, a stereo structure may actively transform from a temporary shape to a permanent shape when the external temperature reaches 160 °C (Fig. 10c)^167^.

A well-established strategy is 3D assembly from 2D precursors driven by compressive buckling, which is invented by Huang et al. ^168–170^. 2D precursors can be fabricated using the most sophisticated materials processes available in state-of-the-art planar technologies. In the assembly process, lithographically defining a set of chemically activated sites followed by transfer printing onto a prestrained elastomer substrate leads to strong covalent bonding at these locations. Releasing the prestrained elastomer creates compressive forces that induce out-of-plane geometric extension. Figure 10d illustrates the major categories of 3D microstructures: 3D filamentary microstructures, 3D mixed microstructures of membranes/filaments and 3D folded microstructures^171^. Notably, the assembly precision is limited due to the use of prestrained elastomers. Theoretical models and process details can be found in the excellent review given by Zhang et al. ^161,171,172^.

### Lab-on polyimide tube

Lab-on polyimide tubes are used to fabricate and integrate microsensors onto polyimide tubes for chemical sensing and biochemical detection applications. These tubes can be manufactured either through precision rolling planar devices into a tube or by surface micromachining on the polyimide tube via 3D photolithography.

**Fig. 11:**
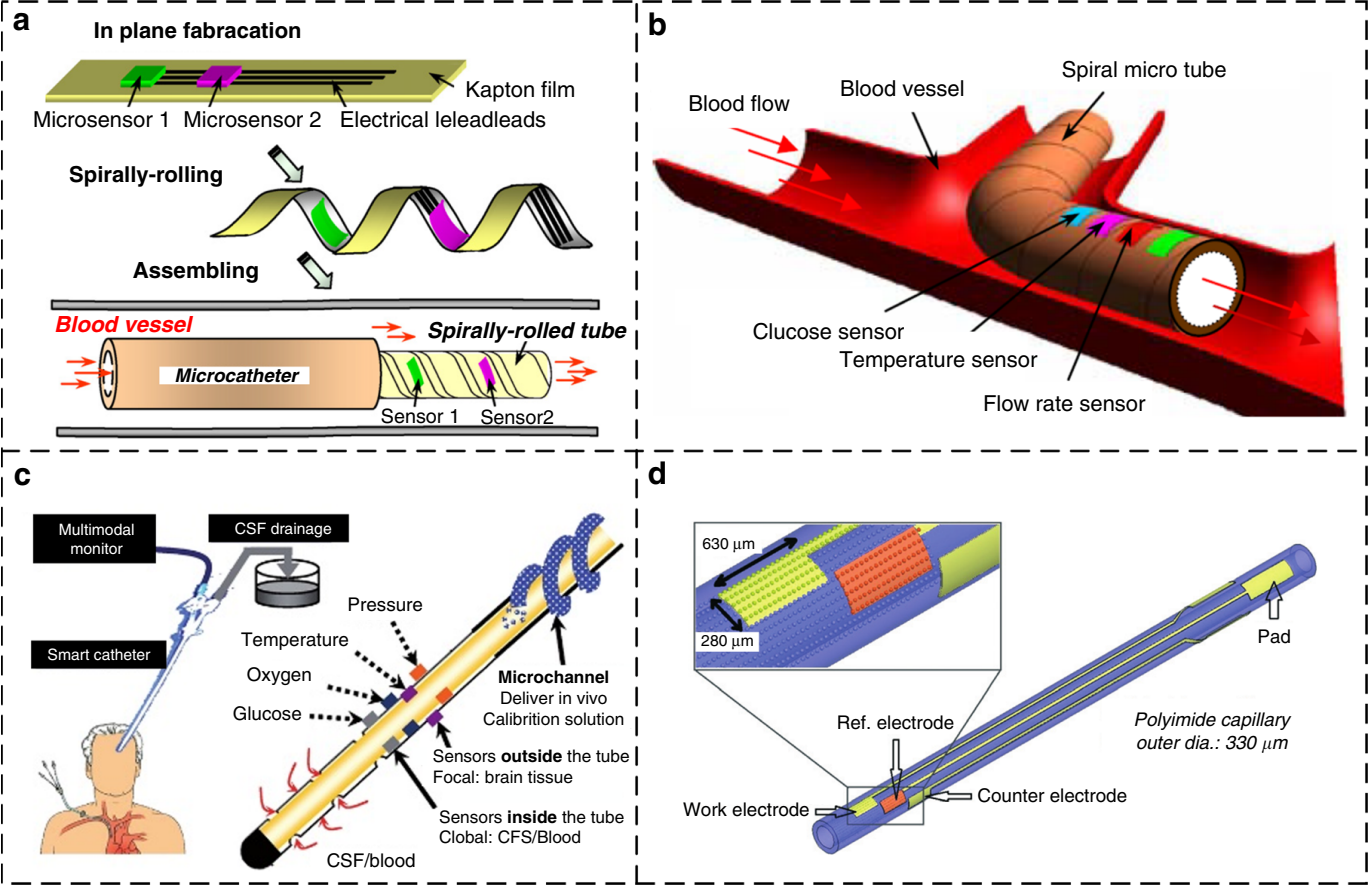
**Lab-on polyimide tube**. **a** Precision rolling planar devices into a tube^173^. **b** Lab-on polyimide tube used for cardiovascular in vivo monitoring^174^. **c** Lab-on polyimide tube used for multimodal neuromonitoring of patients with traumatic brain injuries^175^. **d** Surface micromachining on the polyimide microcatheter curved surface using 3D photolithography^179^

The approach of rolling planar devices into a tube provides a unique manner for assembling multiple sensors on both the inside and outside of the flexible tube for measuring physiological and metabolic data. By using this approach, Li et al. developed various smart microcatheters for the patients’ unique condition^173–178^. The outer diameters of spirally rolled polyimide tubes vary from 700 µm to 1.6 mm (Fig. 11a)^173^. The novel lab-on polyimide tubes integrated with microsensors can be used for cardiovascular in vivo monitoring (Fig. 11b)^174^, the multimodal neuromonitoring of patients with traumatic brain injury (Fig. 11c)^175^, the point-of-care measurements of multiple analytes and many other biomedical applications^176^.

In 2013, Yang et al. successfully developed a UV lithography system with the ±1 µm alignment precision on cylindrical substrates, which can perform multilayer alignment exposure on cylindrical polymer capillary substrates (Fig. 11d)^179^. This 3D photolithography approach is a universal solution for the development of lab-on polyimide tubes. For example, a three-electrode system consisting of two platinum electrodes and one Ag/AgCl reference electrode on a 330-μm-diameter polyimide capillary has been reported for glucose sensor application^180^. A flexible implantable temperature sensor fabricated on a polymer capillary has been designed for monitoring the temperature during microwave hyperthermia^181^. Zhang et al. developed a flexible implantable polyimide catheter with a copper microcoil on the surface for targeted treatment of cardiovascular diseases^182^. By using a 3D photolithography technique, researchers have recently developed a wearable flexible flow sensor for respiratory monitoring in the nasal cavity^183^. The polyimide tube has shown great potential for health monitoring and the preliminary diagnosis of diseases.

## Conclusion and outlook

Widespread emerging applications of polyimide 2D/3D microstructures in the fields of MEMS sensors and flexible electronics provide strong motivation for summarizing well-established microfabrication technologies and for developing new fabrication and assembly approaches. With a focus on polyimide microfabrication, we describe the deposition, patterning, bonding, and assembly techniques and their respective applications. Although the long history of MEMS technology lays a consolidated foundation for polyimide fabrication, new fabrication approaches, such as the EBD of polyimide nanocomposite films, FEP-based polyimide bon ding, and lab-on polyimide tubes, have emerged with innovation in material science and rapid progress in flexible electronics. The technological challenges include the development of high-resolution PSPIs, integration of polyimide into various polymers, improvements in the homogeneity levels in localized LIG and EBD processes, plasma etching of HAR polyimide microstructures, and batch fabrication of 3D polyimide devices.

There is vast opportunity in the development of novel polyimide-based multimodal flexible sensors, microcatheters, and soft robots, requiring continuous progress in polyimide fabrication technologies. From our point of view, one potential technology direction exists in the deposition of functional organic and inorganic materials on polyimide films at moderate temperatures to expand the transduction mechanisms of polyimide-based sensors and actuators. Another important fabrication technique is the 3D or 4D printing of polyimide microstructures with high spatial resolutions, providing a universal method for the development of multifunctional polyimide devices. During the past decades, the integration of silicon-based MEMS with integrated circuits has achieved great success in technological evolution with high-value-added products. In a very similar manner, there is a strong demand and opportunity level for the integration of polyimide sensors with flexible electronics. By standardizing polyimide microfabrication processes and heterogeneous integration technologies with other smart materials, we anticipate a new era of polyimide-based flexible sensors, electronics, and integrated systems.
